# Radiation graft post-polymerization of sodium styrene sulfonate onto polyethylene

**DOI:** 10.1007/s10967-013-2688-3

**Published:** 2013-08-21

**Authors:** Natalia K. Kitaeva, Vladimir R. Duflot, Natalia S. Ilicheva

**Affiliations:** Branch of FSUE “Scientific and Research Institute of Physical Chemistry named after L.Ya. Karpov”, 109 km, Kievskoye Shosse, Obninsk, 249033 Kaluga Region Russia

**Keywords:** Gamma radiation, Graft polymerization, Sodium styrene sulfonate, Acrylic acid, Polyethylene

## Abstract

Post-irradiation grafting of sodium styrene sulfonate (SSS) in the presence of acrylic acid (AA) has been investigated on polyethylene (PE) pre-exposed to gamma radiation at room temperature in the air. Special attention was paid to the effect of low molecular weight salt additives on the kinetics of graft copolymerization of SSS and AA. The presence of SSS links in the grafted PE copolymers was detected by the methods of UV and FTIR spectroscopy. Based on the FITR spectroscopy and element analysis data, a mechanism was proposed for graft copolymerization of SSS and AA onto PE. The mechanical properties of the graft copolymers were studied. It was established that PE copolymers grafted with sulfonic acid and carboxyl groups have higher strength characteristics (16.3 MPa) compared to the samples containing only carboxyl groups (11 MPa).

## Introduction

Modification with ionogenic monomers is known to increase polarity and ion-exchange properties of polyethylene (PE) [1–9]. Radiation graft polymerization of various monomers is commonly used as a simple and efficient method of changing the properties of the matrix polymers. Radiation grafting can be accomplished in a “direct” manner during simultaneous irradiation of monomers and polymers [10–12], or using a post-irradiation method, which can be realized on alkyl radicals [10], peroxides and/or hydroperoxides [13–17] produced on the polymer chains during irradiation in air. The most attractive and commercially viable process is accomplished with involvement of peroxide centers because it is possible to separate a stage of irradiation (formation of initiation centers) from actual grafting.

Post-irradiation grafting is considered promising for commercial production of strong-acid ion-exchange membranes as an alternative to labor-intensive and environmentally vulnerable multi-stage technologies available at present [7, 8, 18–22]. However, numerous efforts failed while preparing strong-acid ion-exchange membranes by post-irradiation grafting of PE with a sulfonic acid group containing monomer—sodium styrene sulfonate (SSS) [23].

Post-irradiation grafting of SSS and acrylic acid (AA) from the binary mixture of monomers onto high density polyethylene (HDPE) (is an interesting approach tried in [24–26]). The authors believed the process would occur on alkyl radicals.

The authors in [27] carried out SSS and AA grafting onto polypropylene matrix pre-irradiated with electrons in the air. The polypropylene fibers thus prepared were chemically active and contained sulfonic acid and carbonyl groups at the same time.

Thus, there exists a principle possibility for post-irradiation grafting of SSS and AA onto PE pre-irradiated at room temperature in the air.

However, in [24–27] no consideration was given to the distribution of SSS links throughout the grafted copolymer thickness, which is most significant for cation-exchange membranes in electrochemical applications. Moreover, changes in physico-mechanical properties of the film or fiber materials during modification were not investigated.

The objective of this work is to study SSS graft polymerization in the presence of AA onto PE pre-irradiated at room temperature in the air, physico-mechanical characteristics and distribution of the grafted copolymer throughout the thickness of the polymer matrix.

## Experimental

### Irradiated polyethylene

PE film of 13 μm thickness was made in accordance with GOST 10354–82 by extrusion of low density polyethylene. The film was irradiated in a cobalt 60 (^60^Co) gamma ray source at room temperature in the air. A dose rate and absorbed dose were 2.5 kGy h^−1^ and 20 kGy, respectively.

### Grafting procedure

Grafting was carried out at 70 ± 1 °C in the aqueous solutions containing AA, SSS and ferrous ions. The concentration of AA in the experiments varied from 4.0 to 10.5 mol dm^−3^; the concentrations of SSS and ferrous ions were 1 and 0.018 mol dm^−3^ [17], respectively. Sodium chloride and sodium acetate were used as additives.

Grafting degree (*g*, %) was determined gravimetrically as the ratio of mass change to the initial mass of the sample

$$ g(\% ) = \frac{{(W_{\text{g}} - W_{1} )}}{{W_{1} }}  \, \times 100, $$

where *W*_1_ and *W*_g_ are the weights of the ungrafted and grafted samples, respectively.

### Measurements

The total concentration of ferrous ions and ferric ions in the solution was determined spectrophotometrically using a sulfosalicylic complex [28], the concentration of peroxide groups in the irradiated PE films was measured by the iodometric method [29, 30].

UV spectra were recorded in the range of wavelengths from 180 to 500 nm (Agilent 8453 spectrophotometer).

FTIR spectra were recorded with a Fourier spectrometer FSM 1202.

The distribution of carbon, oxygen, and sulfur throughout copolymer film thickness was investigated by scanning electron microscopy and tip scanning X-ray microanalysis using a scanning electron microscope JSM-U3 “Jeol” with the energy-dispersive X-ray attachment Win EDS “Getac”.

The mechanical characteristics were measured at room temperature on a universal tensile strength testing machine Zwick 0.05 at the loading rate of 10 mm min^−1^.

## Results

Gamma irradiation of PE in the presence of oxygen and long-term storage result in the formation of peroxides (mainly dialkyl) in PE matrix. The concentration and distribution of peroxides in the volume of the polymer matrix depend primarily on radiation conditions (dose rate, absorbed dose, etc.) [16, 17]. It was established by the chemical analysis that under these radiation conditions the concentration of peroxides in PE was 0.06 mol kg^−1^, and they were homogeneously distributed over the film thickness. Post-irradiation grafting of vinyl monomers onto PE irradiated in the air is initiated by peroxy radicals, which are formed due to heat-induced decomposition of peroxides [31] or reduction in interaction with transition metal salts [17, 32, 33], which is more probable in the case of PE.

In this work ferrous sulfate was used for this objective. The concentration of ferrous ions in the grafting solution was selected on the basis of previous studies and was 0.018 mol dm^−3^ [17].

Radical post-irradiation grafting onto PE irradiated in the air can be schematically presented as a set of the following basic reactions [13, 16, 17]:

1 $$ ({\text{PE}}) \mathop{\longrightarrow}\limits^{{{\text{Irradiation, \; O}}_{ 2} }} ({\text{PE}}) - {\text{OO}} - ( {\text{PE)}} $$

2 $$ ({\text{PE}}) - {\text{OO}} - ( {\text{PE)}} + {\text{Fe}}^{ 2+ } \to ({\text{PE}}) - {\text{O}}^{\bullet} + {\text{Fe}}^{ 3+ } + ( {\text{PE)}} - {\text{O}}^{ - } $$

3 $$ ({\text{PE}}) - {\text{O}}^{\bullet} + {\text{M}} \to ({\text{PE}}) - {\text{O}} - {\text{M}}^{\bullet} $$

4 $$ ({\text{PE}}) - {\text{O}} - {\text{M}}^{\bullet} + {\text{nM}} \to ({\text{PE}}) - {\text{O}} - {\text{M}}_{\text{n}} - {\text{M}}^{\bullet} $$

and corresponding reactions of chain termination.

A resulting graft copolymer contains PE macromolecules with grafted chains.

The difficulty in grafting ionogenic monomers from aqueous solutions onto such hydrophobic polymer as PE is primarily due to lower rate of reaction (3) because the monomer solution does not wet the polymer surface. The second reason is low efficiency of reaction (4) caused by mutual repulsion between ionized groups of the growing polymer radical and a monomer [34].

Two approaches were used to decrease the mutual repulsion of the hydrophobic PE and ionized monomer, as well as the growing polymer radical and a monomer. The preference is given to the use of additional co-monomers with a low degree of ionization of ionogenic groups. AA is a suitable co-monomer for this objective because grafting of AA onto PE is thoroughly studied [13–17]. The use of AA could provide a hydrophilic surface of PE and, as a result, good wetting with strongly ionized SSS molecules. In addition, the co-monomer could decrease the repulsion of non-ionized carboxyl groups and ionized sulfonic acid groups in low-acidic grafting solutions (pK_a_: AA—4.25, organic sulfonic acids—3, polyacrylic acid (PAA)—6.4–6.8, and the value of the apparent dissociation constant of polystyrene sulfonate—0.38 [35, 36]).

Thus, the use of a binary mixture of monomers (AA and SSS) makes it possible to carry out SSS grafting onto PE.

**Fig. 1:**
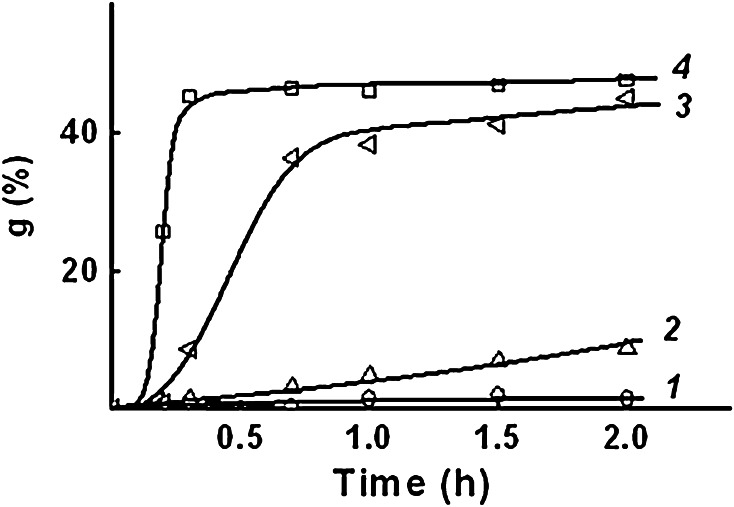
Effect of monomer concentration in the grafting solution on AA grafting kinetics. AA concentration, mole dm^−3^: *1* 4.2, *2* 6.3, *3* 8.4, *4* 10.5

The preliminary studies on kinetics of AA grafting are shown in Fig. 1. It is seen that monomer concentration significantly affects the initial rate of grafting.

The initial rates of AA grafting (Table 1) were calculated from the kinetic data. The values show that with increasing AA concentration in the grafting solution 2.5 times the initial rate of polymerization increases nearly 55 times.

**Table 1:** Effect of monomer concentration in the grafting solution on the initial rate of grafting

Monomer concentration (mol dm^−3^)	Rate of grafting (% h^−1^)
4.2	2.5
6.3	4.7
8.4	73.2
10.5	136.2

**Fig. 2:**
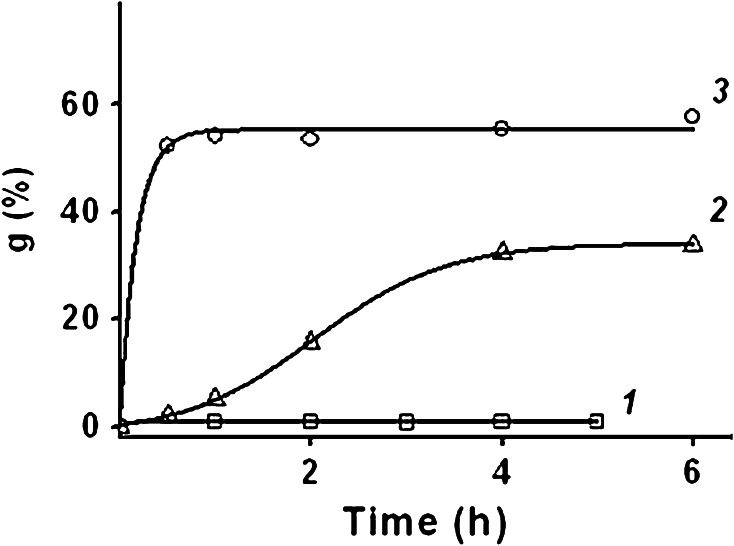
Effect of AA concentration on grafting kinetics. SSS concentration: 1 mol dm^−3^, AA concentration, mol dm^−3^: *1* 0, *2* 6.3, *3* 10.5

The use of a binary mixture of monomers, differing in the degree of dissociation of ionogenic groups, can change the process of grafting. Kinetics of AA and SSS grafting from the binary mixture is shown in Fig. 2. From Fig. 2 it is seen that with increasing AA concentration in the grafting solution the initial rate of the process and grafting degree also increase. Thus, grafting degree reaches 32 % for 4 h grafting at AA concentrations 6.3 mol dm^−3^ and 52 % for 0.5 h grafting at AA concentrations 10.5 mol dm^−3^ (Fig. 2, curves 2, 3).

SSS grafting from the binary mixture of monomers is confirmed by absorption peaks in the regions of 204, 230 and 261 nm of the UV spectra, which correspond to benzene rings in the graft copolymer samples [37]. Similarly, in [38] the emulsion with SSS content exhibits strong absorption in the region of 200–300 nm with the peak at 254 nm, which corresponds to vibrations of benzene ring bonds in the sodium polystyrene sulfonate (PSS) segment of the polymer chain. Thus, UV spectroscopy of the graft copolymers confirms SSS grafting onto PE in the presence of AA in the reaction mixture.

However, it is seen from the UV spectra that SSS grafting is still complicated in the studied conditions, despite the presence of AA in the reaction solution. This is because pH of the solution is 3.1–3.7 at the concentration of AA 4–4.5 mol dm^−3^ and 2–2.3 at 6–6.5 mol dm^−3^, and, therefore, in addition to sulfonic acid groups, carboxyl groups are also ionized, at least, partially, which has an overall negative effect on the efficiency of SSS grafting.

To decrease the influence of electrostatic repulsion between the negatively charged growing radical and the monomer, a method of ion pair formation was used. Due to this approach the charge of carboxyl and sulfonic acid groups is screened, and the mechanism of AA and SSS grafting becomes similar to the mechanism of grafting of uncharged monomers [24, 34].

**Fig. 3:**
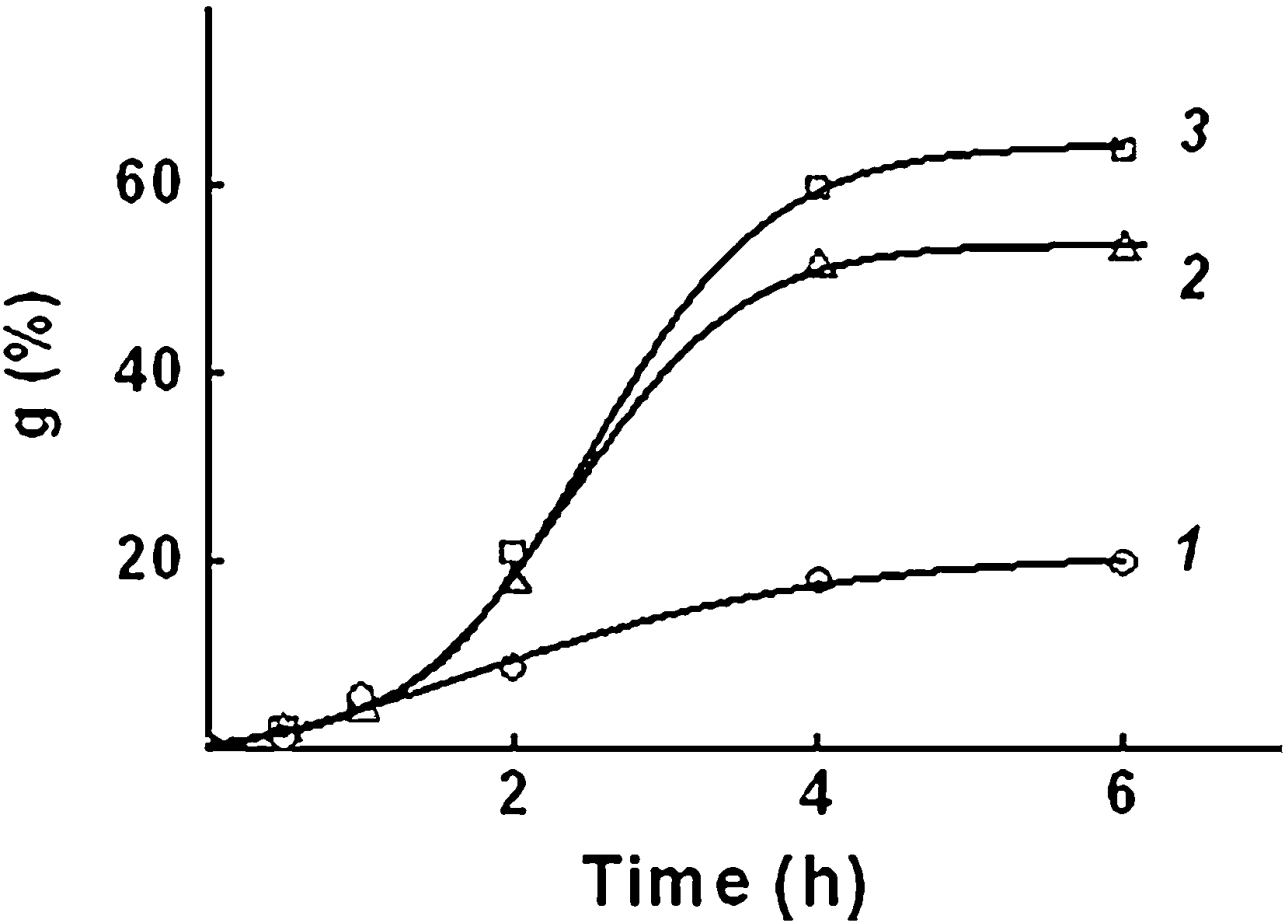
Effect of sodium salt additives on AA grafting kinetics. *1* no additives, *2* CH_3_COONa, *3* NaCl. Concentration, mol dm^−3^: AA—6.3; CH_3_COONa—2.0; NaCl—2.0

**Fig. 4:**
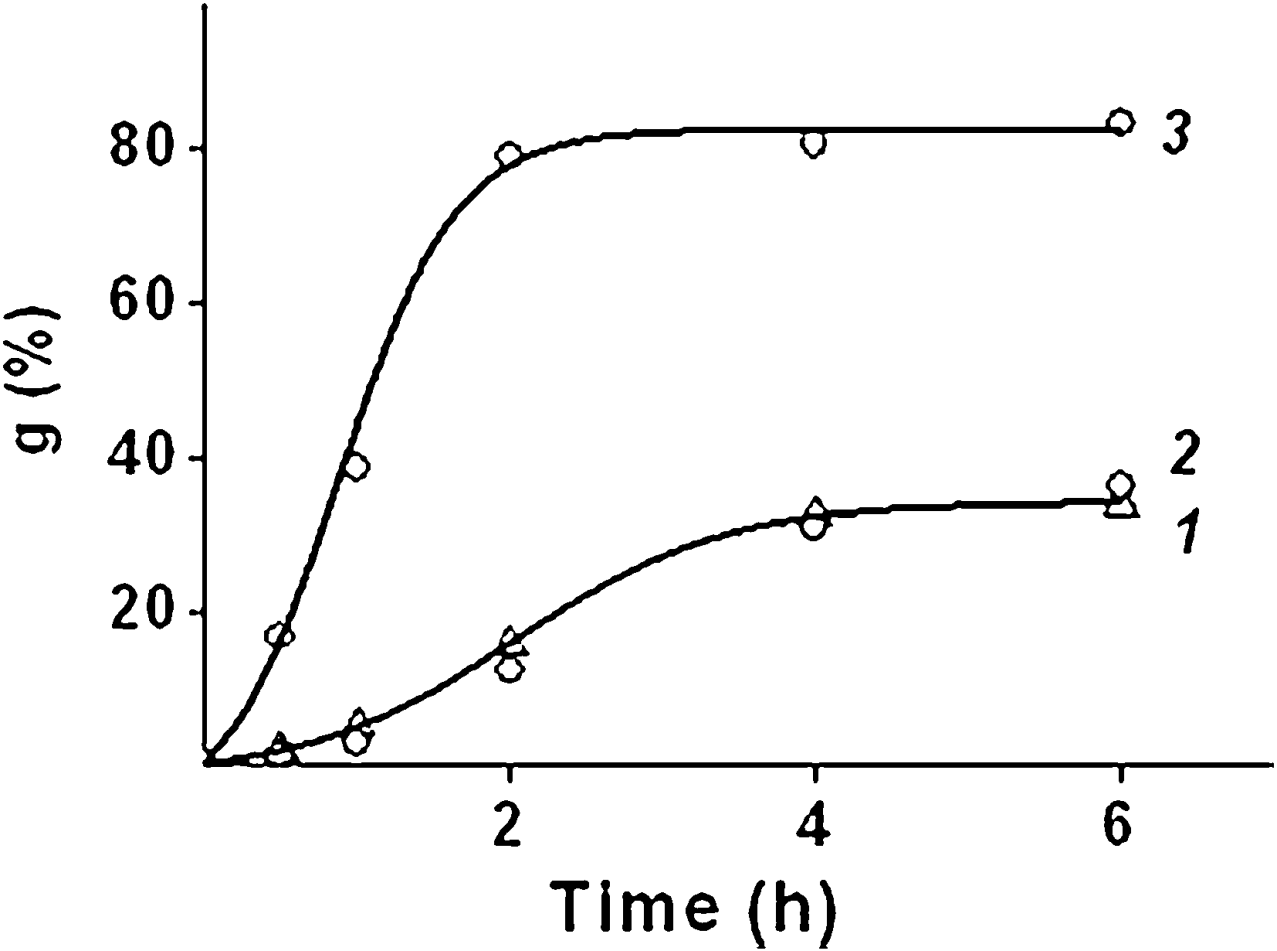
Effect of sodium salt additives on the kinetics of AA and SSS grafting: *1* no additives, *2* CH_3_COONa, *3* NaCl. Concentration, mol dm^−3^: AA—6.3; SSS—1.0; CH_3_COONa—2.0; NaCl—2.0

Low molecular weight sodium salts (chloride and acetate) were added into the reaction mixture to form ionic pairs, and effects of the salt additives on AA graft polymerization (Fig. 3) and graft polymerization of AA and SSS from the binary mixture (Fig. 4) were studied.

From Fig. 3 it is seen that the addition of sodium chloride, as well as sodium acetate into the grafting solution results in significant increase of the rate and maximum grafting of PAA. The maximum of PAA grafting is 1.2 times higher in the presence of sodium chloride as compared to sodium acetate. Dissimilar effects of different sodium salts can be explained by dissociation differences, and, consequently, by a higher content of sodium ions in case of sodium chloride at the same concentration in the solution.

The addition of sodium acetate does not affect graft polymerization of AA and SSS (Fig. 4, curves 1, 2): the initial rate grafting—10 % h^−1^, the maximum degree of grafting—34 %. This is probably due to the fact that styrene sulfonic acid is stronger than acetic acid, and therefore, sodium acetate is a low-dissociating salt in the reaction solution containing SSS.

In case of addition of sodium chloride (Fig. 4, curve 3) the initial rate of grafting of AA and SSS reaches 48 % h^−1^, the maximum degree of grafting—83 %, that in 2.4–2.5 times higher than without additives sodium chloride.

**Fig. 5:**
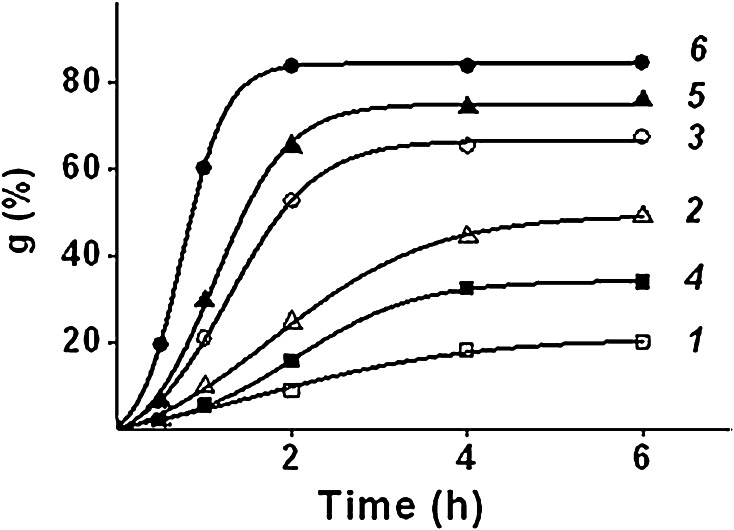
Effect of sodium chloride on the kinetics of AA grafting and of AA and SSS grafting. AA concentration: 6.3 mol dm^−3;^ SSS concentration, mol dm^−3^: *1, 2, 3* 0; *4, 5, 6* 1.0; NaCl concentration, mol dm^−3^: *1, 4* 0; *2, 5* 1.0; *3, 6* 4.0

The effect of sodium chloride concentration on the kinetics of graft polymerization was studied on the samples of PE with grafted PAA (PE-g-PAA) (Fig. 5, curves 1, 2, 3), and PE with grafted copolymer of AA and SSS (PE-g-PAA-co-PSS) (Fig. 5, curves 4, 5, 6).

It can be seen from Fig. 5 that increasing of the sodium chloride concentration in the reaction solution from 0 to 4.0 mol dm^−3^ increases both the initial rate of grafting and maximum grafting.

Herewith, the sodium chloride additive in the reaction solution affects the AA grafting more than the AA and SSS grafting, first of all because of the formation of ion pairs between sulfonic acid groups and sodium ions. The formation of the ion pairs leads to the shielding of the similar charges in the growing polymer chains with the result that the polymerization between ionized monomer and polymeric radical proceeds as if they are non-ionized.

**Fig. 6:**
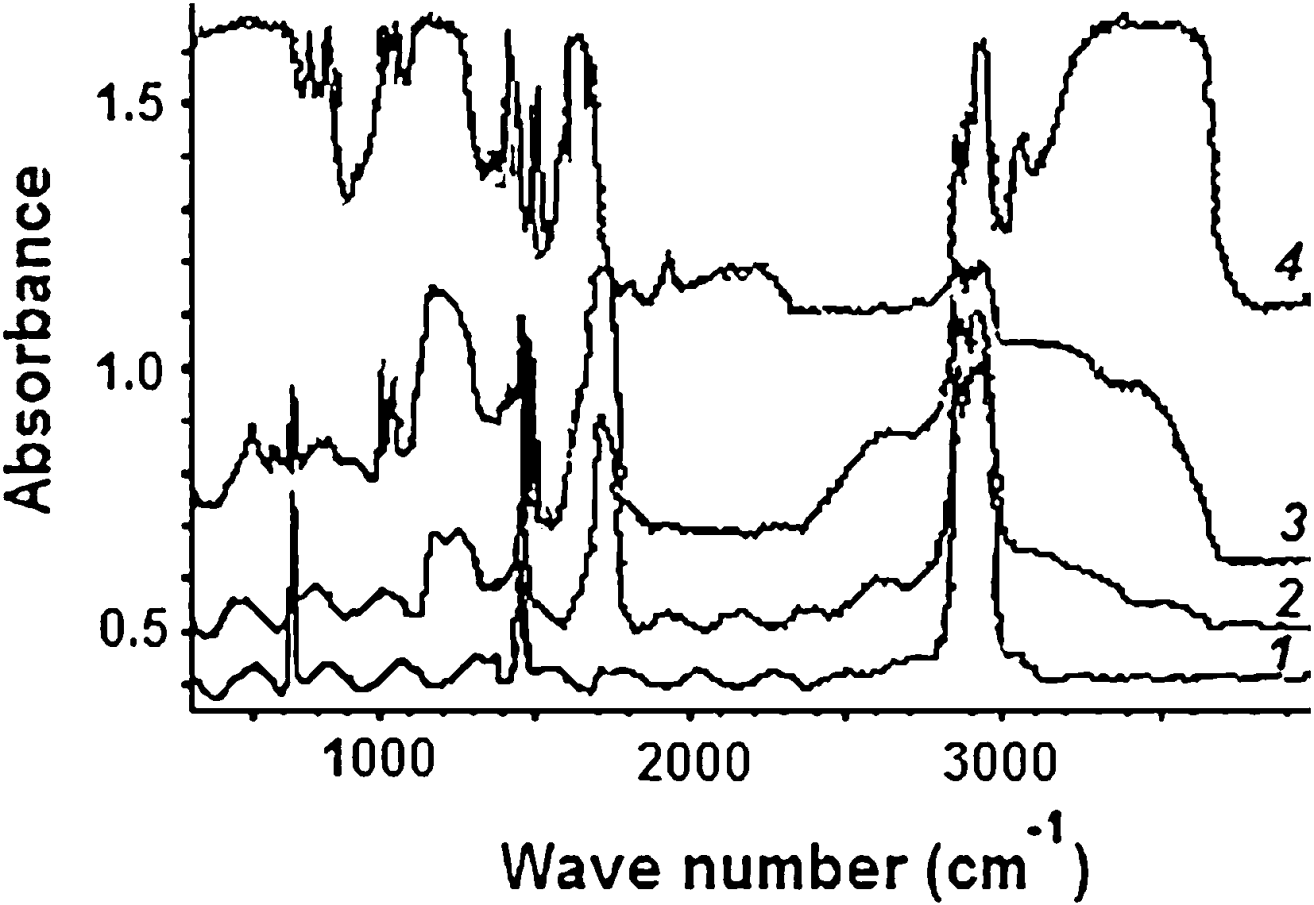
FTIR spectra: *1* PE-g-PAA (*g* = 14.8 %); *2* PE-g-PAA-co-PSS (*g* = 11.8 %); *3* PE–g–PAA-co-PSS (*g* = 82.1 %); *4* PSS

The graft copolymers were analyzed by FTIR spectroscopy. The FTIR spectra of PSS, PE-g-PAA and PE-g-PAA-co-PSS are shown in Fig. 6. It is seen that the FTIR spectra of PSS (Fig. 6, curve 4) have characteristic bands of asymmetric and symmetric vibrations at 1340–1350 and 1150–1160 cm^−1^. The FTIR spectra of PE-g-PAA-co-PSS (Fig. 6, curves 2, 3) include additional peaks at 1637, 1602 and 1496 cm^−1^, which correspond to the stretching vibrations of the C_ar_–C_ar_ bond [39], and the peak at 1411 cm^−1^, corresponding to asymmetric stretching vibrations of S=O bond in sulfonic acid groups. The doublet at 1034 and 1010 cm^−1^ corresponds to the symmetric stretching vibrations of S=O bond in sulfonic acid groups. The doublet at 835 and 776 cm^−1^ corresponds to deformation vibrations of C_ar_–H bonds. The absorption bands in the spectrum of PE-g-PAA-co-PSS coincide with the absorption bands in the PSS spectrum. Similar to [25], the infrared spectra were recorded for non-irradiated PE pre-soaked in the solution containing the monomers (AA and SSS) and polymers (PAA and PSS). The absorption peaks corresponding to C=O and S=O bond vibrations were not recorded in the spectra of non-irradiated PE. Thus, the method of FTIR spectroscopy also confirms SSS grafting onto PE.

To assess the content of the grafted macromolecules in the copolymer, the FTIR spectra were pre-recorded for the reference samples of PAA and PSS homopolymers of pre-set composition. Then the characteristic absorption bands were selected at the frequency of 1040 cm^−1^ and 1722 cm^−1^, which correspond to the stretching symmetric vibrations of S=O bond in the sulfonic acid group and to the stretching vibrations of C=O bond in the carboxyl group, respectively [39–41]. Then the mixtures of known different containing of PAA and PSS were prepared gravimetrically and FTIR spectra were recorded. In addition, a calibration curve was obtained for the ratio D_1722_/D_1040_ optical densities to the ratio of PAA and PSS molar concentrations.

**Fig. 7:**
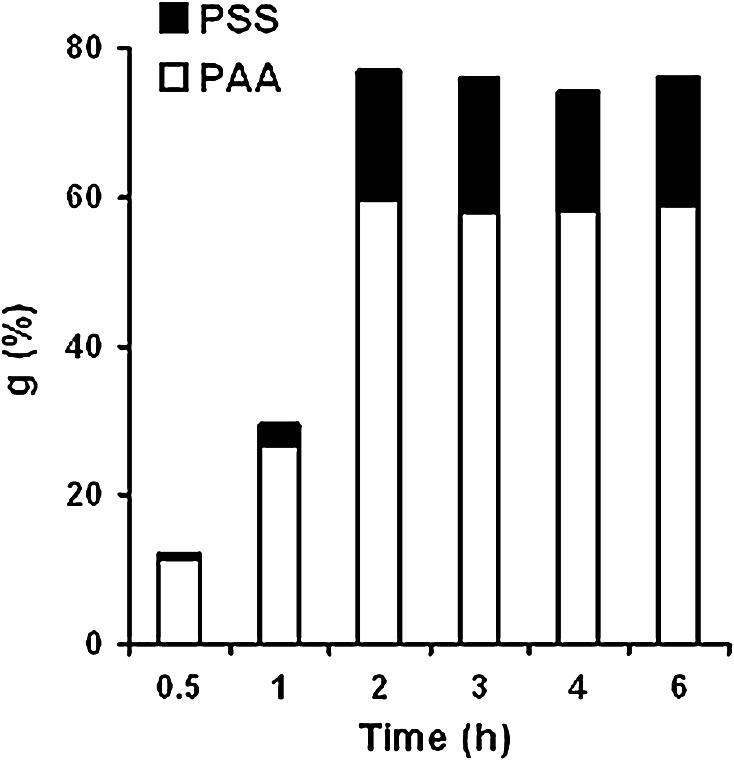
Effect of grafting time on content of the grafted polymer. NaCl concentration: 1.0 mol dm^−3^, concentration, mol dm^−3^: AA—6.3; SSS—1

Based on the analysis of the graft copolymer composition at different times of grafting, it is also possible to make an assumption about the mechanism of AA and SSS grafting onto PE. Thus, Fig. 7 shows the FTIR spectra processed for the kinetics of AA and SSS grafting in the presence of 1.0 mol dm^−3^ sodium chloride (Fig. 5, curve 5). It can be seen in Fig. 7 that SSS grafting is delaying as compared to AA grafting. The grafting of AA and SSS is almost complete in 2 h, but the proportion of grafted SSS is ~23 wt% of the total graft copolymer amount.

**Fig. 8:**
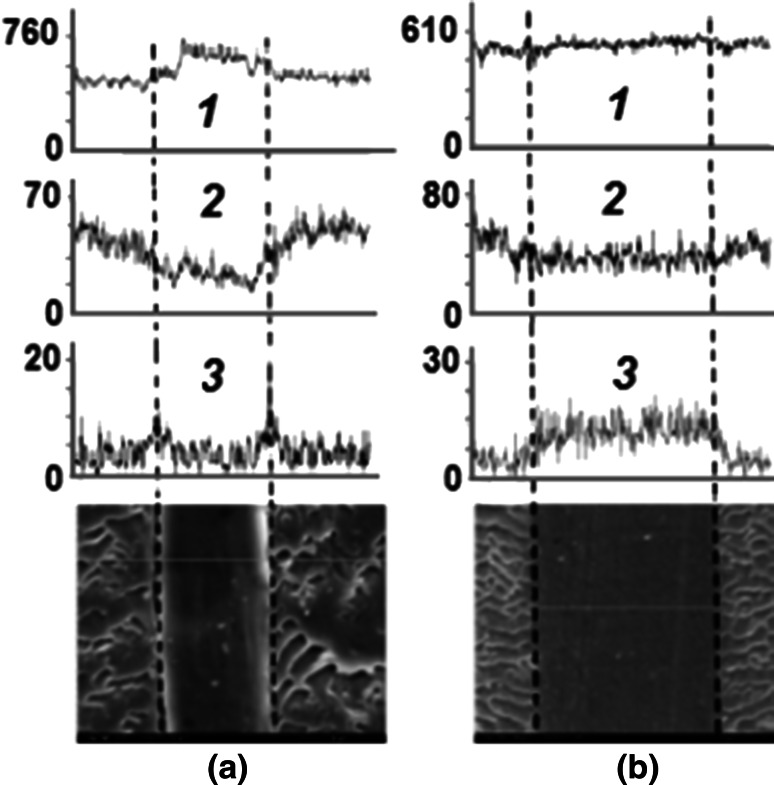
Effect of grafting time on distribution of carbon (*1*), oxygen (*2*) and sulphur (*3*) throughout the thickness of the grafted copolymer. Time of grafting, h: **a** 0.5; **b** 4

A deceleration character of SSS grafting onto PE from the binary mixture of monomers is also confirmed by comparison of element distribution (carbon, oxygen, sulphur) throughout the copolymer thickness after 0.5 h and 4 h of AA and SSS grafting (Fig. 8).

It can be seen from Fig. 8a that after 0.5 h AA and SSS grafting onto PE, oxygen is concentrated mainly on the sample surface, and sulfur content in the grafted copolymer is negligible. In 4 h AA and SSS grafting, sulphur appears in the sample composition in addition to oxygen, which indicates the presence of the grafted PSS in the copolymer (Fig. 8b).

Thus, it can be concluded that only AA is grafted from the binary mixture of monomers at the initial stage of the process, and grafted PAA is mainly localized on the surface of PE. Further grafting results in increasing PAA content and appearance of the grafted PSS in the copolymer, which correlates well with the FTIR spectroscopy data.

Dependence 2 and 3 (Fig. 8b) shows that the content of oxygen and sulphur is uniformly on the surface and inside the film after 4 h of grafting. Thus both PAA and PSS are homogeneously distributed throughout the thickness of the grafted copolymer with increasing grafting time.

According to [19, 24, 26, 27], the presence of sulfonic acid groups positively affects water absorption ability of the graft copolymer. As a consequence, the degree of swelling changes and cation exchange properties increase. On the other hand, the same groups can decrease such properties of graft copolymers as mechanical strength, which is important for practical applications. Moreover, the mechanical properties depend equally on the total content and distribution of sulfonic acid groups on the surface and in the bulk of the graft copolymer. However, the data on distribution and effect of sulfonic acid groups on the mechanical properties during AA and SSS grafting from the binary mixture can be hardly found in scientific publications.

Investigations of the mechanical properties showed that copolymer strength did not change with the time of AA grafting onto PE in the absence of SSS. However, with SSS incorporation to the monomer mixture a sharp increase of the graft copolymer strength was observed in 2 h. The value of tensile strength increased from 10.2 MPa for the initial PE to 16.3 MPa for the copolymer with grafted PAA and PSS.

Elasticity studies of the graft copolymers showed an extreme dependence on the time of polymerization. After 2 h of grafting elastic properties drastically decrease, and in 4 h the elasticity is 64.0 % for copolymers with grafted PAA and 21.5 % for the copolymer with grafted PAA and PSS.

This effect of ionogenic monomers on the mechanical properties of grafted copolymers PE-g-PAA-co-PSS is attributed foremost to their frontal character of PE grafting. Starting from the surface of PE film, fronts of grafting penetrate deep into PE sample so grafted copolymer has a higher flexibility in almost constant strength characteristics. After 2 h of grafting fronts getting close, grafting is ended and more homogeneous system, consisting of PE and uniformly distributed through its thickness grafted macromolecules, is appear. It can be proved by the presented above data obtained from the FTIR spectra (Fig. 7) and by elemental analysis (Fig. 8).

After 2 h of grafting the effect of grafted PAA or grafted PAA–PSS on the mechanical properties becomes the dominant. This is increases the strength of the graft copolymer and reducing their elasticity, because unlike PE, PAA and PSS are glassy polymers [42–44].

Thus, 2 h of grafting is the critical point wherein specify system properties (mechanical, electrical, swelling etc.) are changed dramatically.

Physical and chemical properties of PE copolymers with grafted PAA and PSS were comparable with the values of PSS swelling in aqueous solution, cation exchange capacity and ionic conductivity given in [24–27].

## Conclusions

Thus, the studies of post-irradiation graft polymerization of SSS onto PE in the presence of AA experimentally proved a possibility of grafting highly ionized SSS monomer onto PE pre-exposed to gamma-irradiation at room temperature in the air.

It was established that graft polymerization of SSS and AA from the binary mixture onto PE has a delaying nature.

In addition to confirm the presence of SSS links in the graft copolymer the methods of UV and FTIR spectroscopy and element analysis were used. They also demonstrated that SSS is grafted not only on the surface of PE, but also inside the polymer matrix.

## Abbreviations

AA: Acrylic acid
g: Grafting degree
HDPE: High density polyethylene
PAA: Polyacrylic acid
PE: Polyethylene
PE-g-PAA: Polyethylene with grafted polyacrylic acid
PE-g-PAA-co-PSS: Polyethylene with grafted copolymer of acrylic acid and styrene sulfonic acid
PSS: Sodium polystyrene sulfonate, polystyrene sulfonic acid
SSS: Sodium styrene sulfonate
